# Blockade of Adenosine A2b Receptor Reduces Tumor Growth and Migration in Renal Cell Carcinoma

**DOI:** 10.7150/jca.31245

**Published:** 2020-01-01

**Authors:** Ye Yi, Yihong Zhou, Xi Chu, Xiaoping Zheng, Deng Fei, Jun Lei, Huiyue Qi, Yingbo Dai

**Affiliations:** 1 Department of Urology, The Third Xiangya Hospital of Central South University, No.138 Tongzipo Road, Changsha 410600, China.; 2 Department of Urology, The fifth affiliated hospital Sun Yat-sen University, No.52 Meihua Dong Road, ZhuHai 519000, China.

**Keywords:** Adenosine A2b Receptor, MRS1754, MAPK/JNK signal pathway, Renal cell carcinoma.

## Abstract

Adenosine A2b receptor (A2bR) is a member of the G protein-coupled receptor superfamily members, which has been considered involved in the pathogenesis of various cancers. However, little is known about the role of A2bR renal cell carcinoma (RCC). The A2bR expression levels in RCC 769-P and Caki-1 cell lines compared with HK-2 were analyzed by qRT-PCR. 769-P and Caki-1 cells were transfected with shRNA-A2bR to knock down the expression of A2bR. Cell proliferation was detected by MTT assays and colony formation assays. Wounding healing assays and transwell assays were used to evaluate the effects of A2bR on cell capacity of invasion and migration. Finally, potential mechanisms involved in A2bR blockade's effects on altered tumor behaviors were evaluated by western blotting. We showed that A2bR were significantly up-regulated in RCC cells compared to HK-2 cell. Functionally, MRS1754, a selective A2bR antagonist, and knocking-down the expression of A2bR by shRNA reduced proliferation and migration *in vitro* and tumor growth *in vivo*. Furthermore, we demonstrated that A2bR blockade inhibited tumor progression in part via the MAPK/JNK pathway. **Conclusion**: Our findings suggest the A2bR potentially plays an important role in RCC progression and A2bR blockade may be a promising candidate for therapeutic intervention for renal cell carcinoma.

## Introduction

Adenosine is a purine nucleoside which has been described as an important regulator of immune responses in cancers and inflammatory disorders. In the extracellular space, the ectonucleotidases CD39 and CD73 catalyze ATP and ADP into AMP, and AMP to adenosine, respectively 1. Extracellular concentration of adenosine is dependent on the cellular metabolic demand and/or oxygenation status 2. Under the normal condition, extracellular adenosine concentrations are found to be in the range of 10-100nM 3. However, in tumor microenvironment, where hypoxia increases the expression of CD39 and CD 73 leading to an increase in adenosine production, and induces inhibition of adenosine kinase resulting in decreasing adenosine removal, the concentration of adenosine is significantly increased (10-20 fold higher than normal situation) 4-6. It was reported that the enhanced accumulation of adenosine in tumor area may play an important role in regulation of tumor growth and metastasis 7-9.The biological functions of adenosine are mediated via four G protein-couple adenosine receptors (ARs): A1, A2a, A2b, and A3 10. While A1, A2a and A3 adenosine receptors are high-affinity receptors, adenosine A2b receptor (A2bR) has low affinity for adenosine binding 11. Thus, unlike the other adenosine receptor subtypes, the adenosine A2b receptor is only activated by high concentrations of adenosine (under pathophysiological conditions), not by physiological levels 12, 13. Therefore, A2bR may play a role in pathophysiologic conditions associated with massive adenosine release, such as in a tumor microenvironment 14. Studies have reported that A2bR has been involved in the tumor progression of bladder, colon, and prostate carcinoma 15-17. Wei et al. 17 showed A2bR blockade inhibited growth of prostate cancer cells. Similarly, a study from Wang 18 reported that knocking down the expression of A2bR suppressed the tumor growth and promoted the apoptosis in gastric cancer. Mittal et al reported that inhibition of adenosine A2b receptor decreases metastasis in melanoma and triple-negative breast cancer 19.

Renal cell carcinoma (RCC) is a common tumor in urologic system, accounting for approximately 3% of cancers in adults as well as 85% of all primary malignant kidney tumors 20. Until now, although surgical approach is the main treatment for localized RCC, some patients experience postoperative metastasis and locally advance or metastasis at initial diagnosis, which limits the survival of patients 21. As a typical solid tumor, renal cell carcinoma is highly vascularized. However, ischemia and hypoxia are commonly observed in tumor microenvironment, which indicates high concentration of adenosine. To investigate the expression status and function of A2bR in renal cell carcinoma cells may be promising.

In this study, we first revealed that the expression of A2bR was higher in RCC cell lines 769-P and Caki-1 compared with HK-2. Next, we comprehensively showed that the pharmacological and genetic blockade of A2bR, which were mediated through MRS1754 and shRNA-A2bR, reduced cancer cells proliferation, migration, invasion *in vitro* and tumor growth *in vivo*. Collectively, high expression of A2bR on RCC cell provides us new understandings of the role of adenosine receptor in tumor progression and blockade of adenosine A2b receptor may act as a promising candidate for therapeutic intervention for renal cell carcinoma.

## Material and Methods

### Cell culture

In this study, the human renal cell carcinoma cell line 769-P (primary renal carcinoma cell), Caki-1(metastatic renal carcinoma cell) cell lines and the renal tubular epithelial cell line HK-2 were purchased from the Shanghai institute of Cell Biology, Chinese Academy of Science (Shanghai, China). 769-P and HK-2 was cultured in DMEM medium (HyClone, USA) supplemented with 10% fetal bovine serum (FBS) (HyClone, USA). Caki-1 was cultured in RPMI-1640 medium with 10% fetal bovine serum (FBS) (HyClone, USA). And both of them are cultured at 37℃ in a humidified 5% CO2. N-(4-cyanophenyl)-2-[4-(2, 3, 6, 7-tetrahydro-2, 6-dioxo-1, 3-dipropyl-1H-purin-8-yl)-phenoxy] acetamide (MRS1754), a selective antagonist ligand of A2b adenosine receptors, and 2-[6-amino-3, 5-dicyano-4-[4-(cyclopropylmethoxy) phenyl] pyridin-2-yl] sulfanylacetamide (Bay60-6583), a selective A2bR agonist, were obtained from Sigma Chemical (St Louis, MO, USA), dissolved in dimethyl sulfoxide (DMSO). The medium was replenished every day.

### Quantitative real-time PCR

Total RNA was extracted from cells using TRIzol reagent (Invitrogen, Carlsbad, CA, USA) according to the manufacturer's protocol. RNA concentration was quantified using absorbance at 260 nm and the ratio of the absorbance at 260 and 280 nm was used to assess the Bio-Rad PCR system S1000 (Bio-Rad, Hercules, CA, USA). Quantitative real-time PCR was performed using the SYBR Green PCR kit and β-actin was used as the internal control. Specific PCR primers were designed as listed in table 1. All data for the Real-Time PCR was analyzed using the Applied Biosystems 7500 System SDS Software using the standard curve method.

### Lentivirus-mediated A2bR RNA interference

For short hairpin RNA (shRNA) experiments, Lentivirus encoding shA2bR were designed and synthesized by Shanghai GenePharma Co. Two short hairpin RNA oligonucleotides (shRNA 1: 5'-GAGCTCCATCTTCAGCCTTCT-3'; shRNA 2: 5'-GGCATCGGATTGACTCCATTC-3') and a nonsense shRNA (NC: 5'-TTCTCCGAACGTGTCACGT-3') were designed. Stable transfectants were selected with puromycin (2 mg/mL) and verified by quantitative real-time PCR analysis and western blotting.

### Western blotting

Protein extracted from cells were separated by 10% SDS-PAGE and transferred to PVDF membranes (Millipore, Germany). Membranes were blocked and then incubated with primary antibodies. The free protein binding sites were blocked by incubating with PBS containing 5% skimmed milk at 4℃ overnight. After the blot was washed several times, it was incubated with goat anti-rabbit IgG in the blocking solution for 1h.Then, the immunocomplexs were visualized by enhanced chemiluminescence (SuperSignal Pierce Biotechnology). β-actin was used as the loading control. The following JNK (9252), p-JNK (#4668) and β-actin (#12620) were purchased from Cell Signaling technology. The antibody against A2bR (ab40002) was obtained from Abcam.

### MTT assay

769-P and Caki-1 cells were plated and treated in 96-well plates at 2000 cells/well. 769-P and Caki-1 cells were treated with MRS1754 (from 10nM to 350nM) for 48hours with/without the treatment of Bay 60-6583 (10nM). The shRNA group and NC group were seeded onto 96-well plates at a density of 800 cells per well. During a 5-d culture periods, 20μl MTT-medium mixed solution was added to cell culture per day, and incubated for 4 h. After the formazan sediment was dissolved using 150μl DMSO, the absorbance at 490nm was measured on ELASA reader. All experiments were performed in triplicate.

### Colony formation assay

769-P and Caki-1 cells were seeded into 6-well plates (600 cells per well). MRS1754 (200nM and 300nM) was added to the well with/without Bay60-6583 (10 nM). After culturing for 12-14 days, proliferation colonies were stained with crystal violet, and colonies containing more than 50 cells were counted. The control group or cells transfected with shRNA were seeded in 6-well culture plate with 600 cells per well and cultured for 10-12 d. Then the cells were stained with Crystal violet. All experiments were performed in triplicate.

### Wound healing assay

769-P and Caki-1 cells were plated into 6-well plates and maintained to confluence. The wounds were scratched using pipette tips. After that, MRS1754 (200nM and 300nM) was added to the well with/without treatment of Bay60-6583 (10 nM). Images were taken immediately and at 18 h after wounding. After transfected with shRNA, 769-P and Caki-1 cells were plated into 6-well plates and grown to confluence. Wounds were created and photomicrographs were also taken immediately and at 18 h after wounding. The acreage of cell-free area was measured, and the percentage of migration was considered as the ratio of the reduction of acreage to the initial acreage of scraped area (0 h). All experiments were performed in triplicate.

### Cell migration assay

In the migration assays, a 24-well transwell plate (lower chamber) and transwell inserts (upper chamber) with 8μm pore size polycarbonate membrane (Corning, USA) were used. 769-P, Caki-1 cells and the cells transfected with shRNA were cultivated with serum-free culture medium for 24 hours. Then, 769-P and Caki-1 cells in 100μL serum-free medium with different concentrations of MRS1754 (200nM and 300nM) or equal amount of DMSO (control) were added into upper chambers, and the cells transfected with shRNA were also added into upper chambers with serum-free medium. Meanwhile the lower chambers was filled with 700μL medium containing 10% FBS. After 48 h of incubation, tumor cells in the upper chamber were removed carefully. The cells that migrated through the membrane were stained with 0.1% crystal violet and counted.

### Xenograft assay in nude mice

After a week of acclimatization, shRNA-A2bR 769-P and Caki-1 cells as well as shRNA-nonsense cells as NC group(5×10^6^) were injected subcutaneously into the right flank of 6-week-old BABL/c nude mice (n = 5 for each group). Tumor volumes were calculated as width^2^×length/2. On day 40th, the mice were sacrificed and the tumor tissues were removed. The animal protocol used in this study was approved by the Animal Ethics Committee of Central South University.

### Statistical analysis

The statistical analysis was conducted by using SPSS 19.0 software (SPSS Inc., Chicago, IL, USA). The data were expressed as the mean± SD. The Student unpaired t test was used to analyze intergroup differences for two groups, and one-way ANOVA was used to analyze more than two groups. P value < 0.05 was considered to be statistically significant.

## Results

### A2bR is over-expressed in RCC cells and shRNA-A2bR knocks down the expression of A2bR

**Figure 1:**
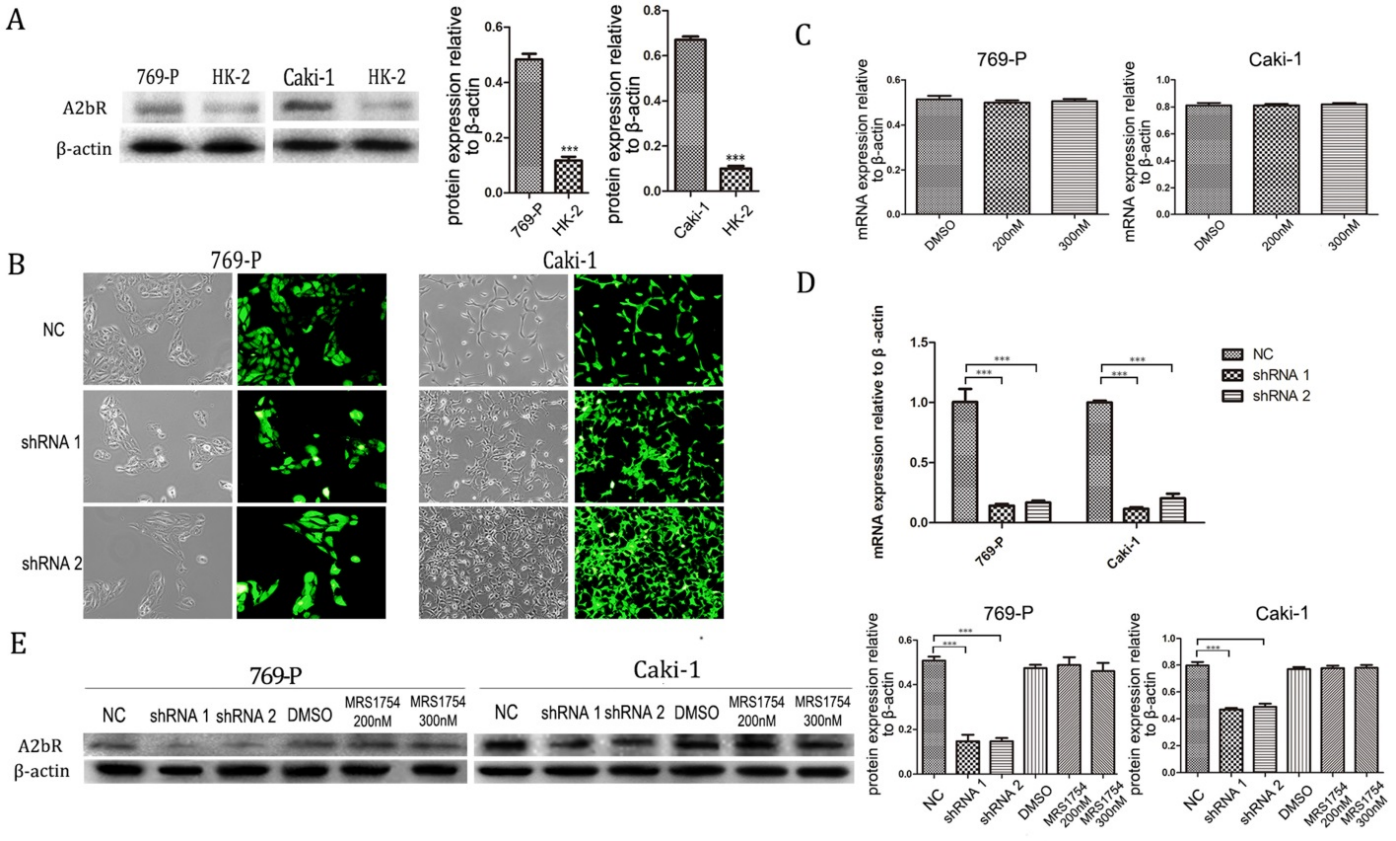
A2bR was relatively high expressed in renal cell carcinoma cell lines. (A) qRT-PCR showed that A2bR in 769-P and Caki-1 cell lines is over-expressed than HK-2 cells. (B) The representative images of shRNA-A2bR transfected cells. (C and E) qRT-PCR results showed that MRS1754 had no significant effects on expression of A2bR of cell lines. qRT-PCR (D and E) results showed that transfection of shRNA-A2bR knocked down the mRNA and protein expression of adenosine A2b receptor. ***p<0.001, **p<0.01, *p<0.05.

Firstly, we examined the expression patterns of A2bR in renal cell carcinoma cell lines (769-P and Caki-1) compared with HK-2 cell. It was shown that the expression of A2bR in 769-P and Caki-1 cells was significantly higher than HK-2 (Fig. 1A; *p*<0.001). To determine the relationship of adenosine receptor expression and effects of MRS1754, we examined the A2bR expression profile of RCC cells after treating with MRS1754 (200nM and 300nM) for 5 days. Then, the mRNA expression level of A2bR was evaluated, and the results showed that the mRNA expression of A2bR was not affected by MRS1754 significantly (Fig. 1C). Similarly, the protein level of A2bR was not affected by MRS1754 (Fig. 1E).These findings suggested that adenosine A2b receptor may play an important role in biological behavior of renal cell carcinoma.

To further explore the impact of A2bR on RCC cells, short hairpin RNAs (shRNA 1, shRNA 2 and a nonsense shRNA) were used to knock down A2bR expression in 769-P and Caki-1 cell lines. After transfected with shRNA (MOI=20) and selected with puromycin (2 mg/mL), up to 90% cells expressed green fluorescent protein (GFP) (Fig. 1B). Western blot and PCR analysis were performed to assess the efficiency of A2bR knockdown compared with shRNA-nonsense group. As shown in Fig. 1D, A2bR mRNA expression in cells transfected with shRNA-A2bR was significantly lower than in NC group (shRNA-769-P cell lines: 0.14 ± 0.015, 0.16 ± 0.017 and 1.00 ± 0.11, shRNA 1, shRNA 2 and NC group, respectively) (shRNA-Caki-1 cell lines: 0.12 ± 0.014, 0.20 ± 0.039 and 1.00 ± 0.014, shRNA 1, shRNA 2 and NC group, respectively). As shown in Fig. 1E, protein expression of A2bR in cells transfected with shRNA-A2bR also was significantly reduced compared NC group (shRNA-769-P cell line: 0.15 ± 0.04, 0.15 ± 0.02 and 0.51 ± 0.02, shRNA 1, shRNA 2 and NC group, respectively) (shRNA-Caki-1 cell line: 0.47 ± 0.02, 0.49 ± 0.03 and 0.80 ± 0.04, shRNA 1, shRNA 2 and NC group, respectively).

### A2bR blockade inhibits cell proliferation *in vitro*

**Figure 2:**
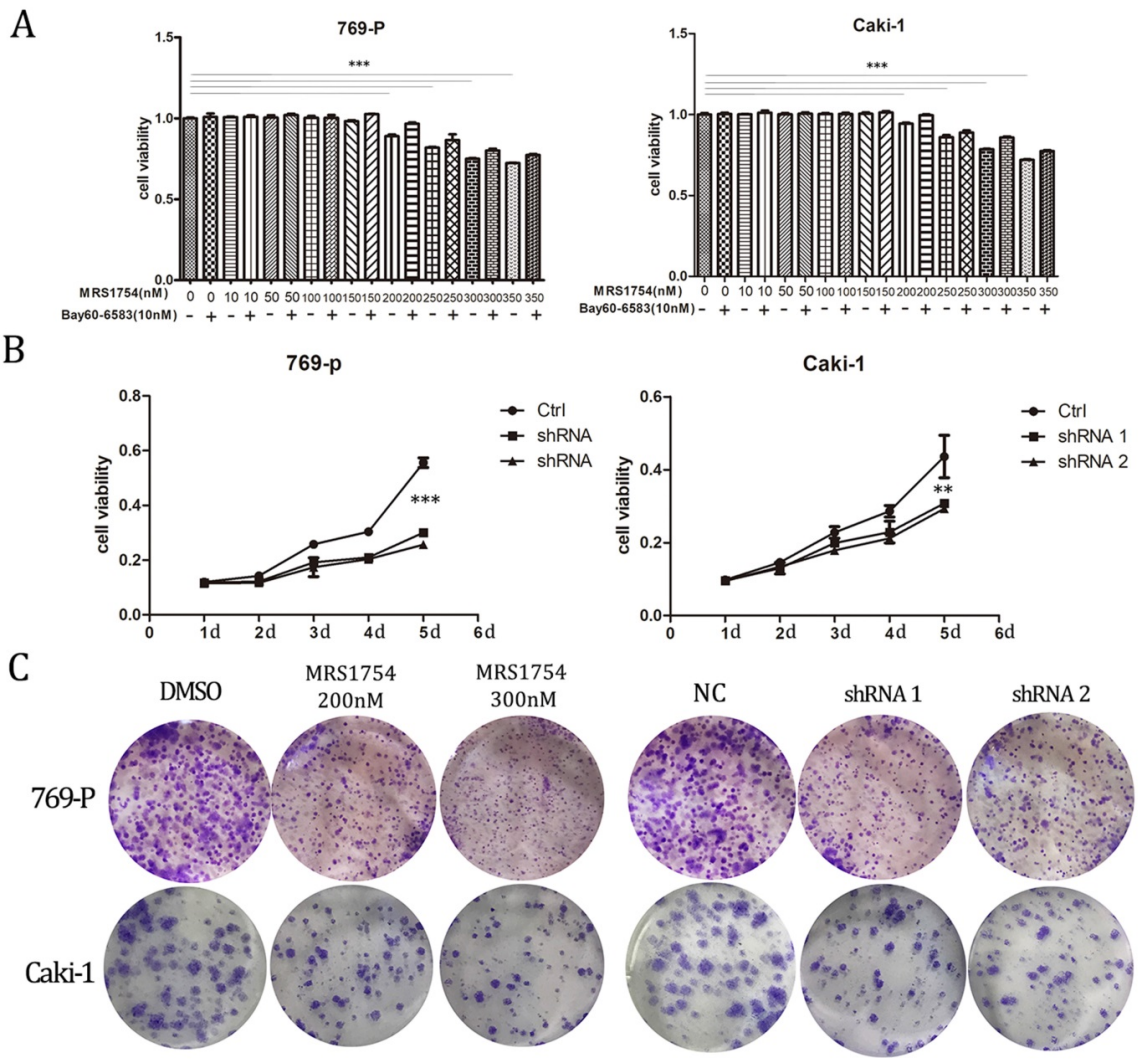
A2bR blockade inhibited proliferation of RCC 769-P and Caki-1 cell lines. MRS1754 inhibited proliferation of 769-P and Caki-1 cell lines evaluated by MTT assays (A) and colony formation assays (C), which could be rescued by Bay60-6583 significantly. Down-regulation of A2bR expression inhibited cell proliferation of RCC cells as detected by MTT (B) and colony formation assays (C). ***p<0.001, **p<0.01, *p<0.05.

After determining that A2bR is over-expressed in RCC cells and establishing the A2bR knockdown cells, we explored the impact of A2bR on RCC cells proliferation by MTT assays. 769-P and Caki-1 cells were cultured in medium with fetal bovine with/without treatment of Bay60-6583(10nM), a selective A2bR agonist, which at the concentration of 10nM didn't affect the cell viability (The results aren't shown). The results showed that MRS1754 reduced renal cell carcinoma cell proliferation in a concentration-dependent manner and Bay60-6583 could rescue the inhibitory effects in cells viability. 769-P and Caki-1 cells viability were significant declined when concentration of MRS1754 was 200nM or greater (Fig. 2A; *p*<0.05). Meanwhile, our results also showed that the shRNA-A2bR group, in which the expression of A2bR was knocked down, had a significant decrease in proliferation of 769-P and Caki-1 cells as compared with NC groups (Fig. 2B; *p*<0.05). Similarly, colony formation capacity was reduced following A2bR blockade (Fig. 2C; *p*<0.05). These data indicated that pharmacological and genetic blockade of A2bR could inhibit 769-P and Caki-1 cells growth *in vitro*.

### A2bR blockade inhibits cell migration and invasion

**Figure 3:**
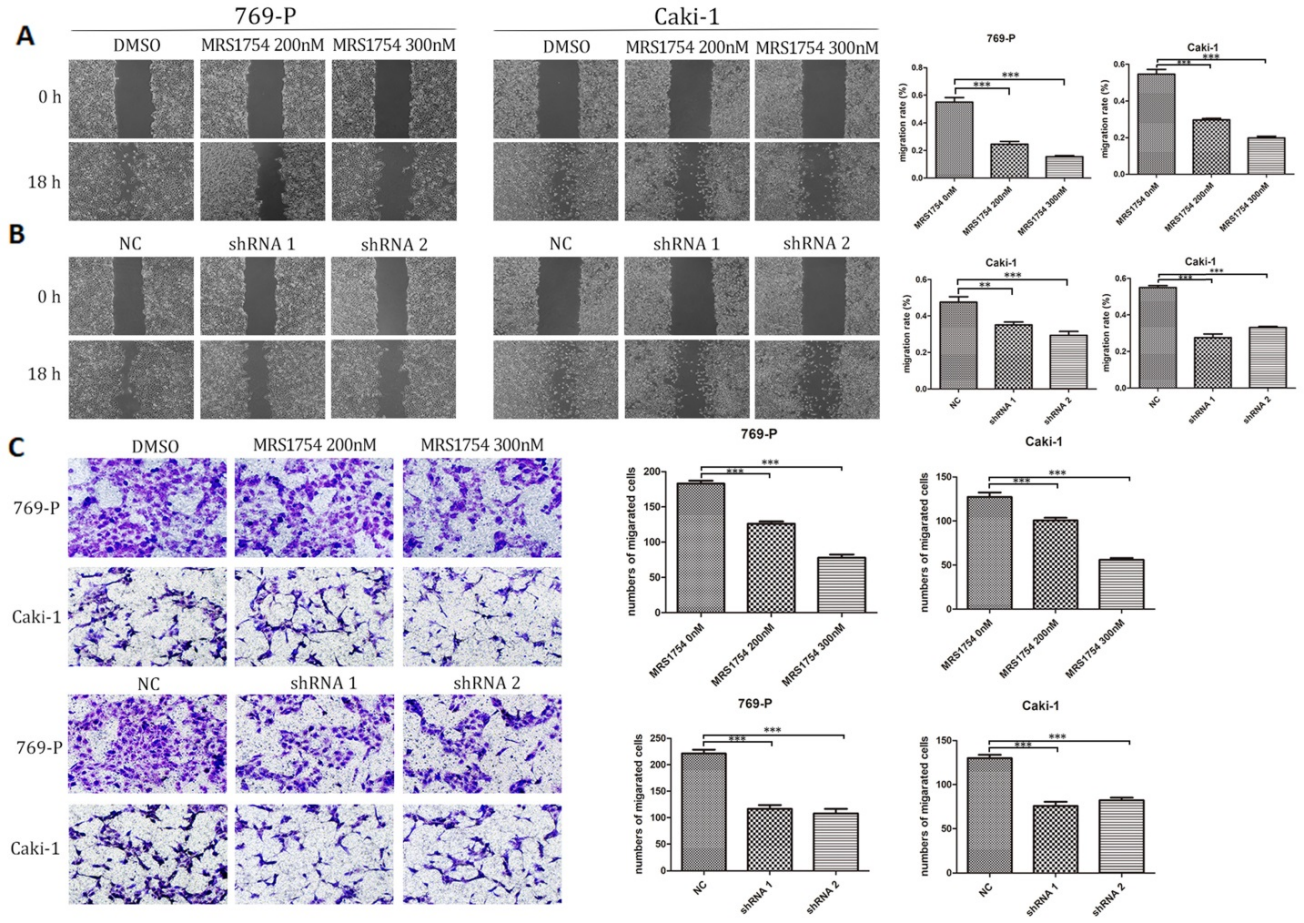
A2bR blockade inhibited cell migration and invasion of RCC cells. (A) MRS1754 inhibited RCC cells migrating into the scratching area in a dose-dependent manner measured by wound healing assays. (B) Suppression of A2bR inhibited 769-P and Caki-1 cells migrating into the scratching area. (C) Transwell assays showed that A2bR blockade reduced 769-P and Caki-1 cells invasion ability. ***p<0.001, **p<0.01, *p<0.05.

Next, we examined the effects of A2bR on migration of 769-P and Caki-1 cells using wound healing assays. The results showed that MRS1754 significantly slowed down the rate of migration in concentration-dependent manners (Fig. 3A; *p*<0.05). RCC cells with shRNA-A2bR transfection also exhibited a lower degree of wound closure compared NC group cells (Fig. 3B; *p*<0.05). In addition, transwell assays showed an obvious decrease in migration capability of 769-P and Caki-1 cells after blockading A2bR (Fig. 3C; *p*<0.05). These results suggested that A2bR blockade could weaken the ability of migration and invasion capability in 769-P and Caki-1 cells.

### Bay60-6583 rescues the inhibitory effect of MRS1754 on RCC cells growth and migration

**Figure 4:**
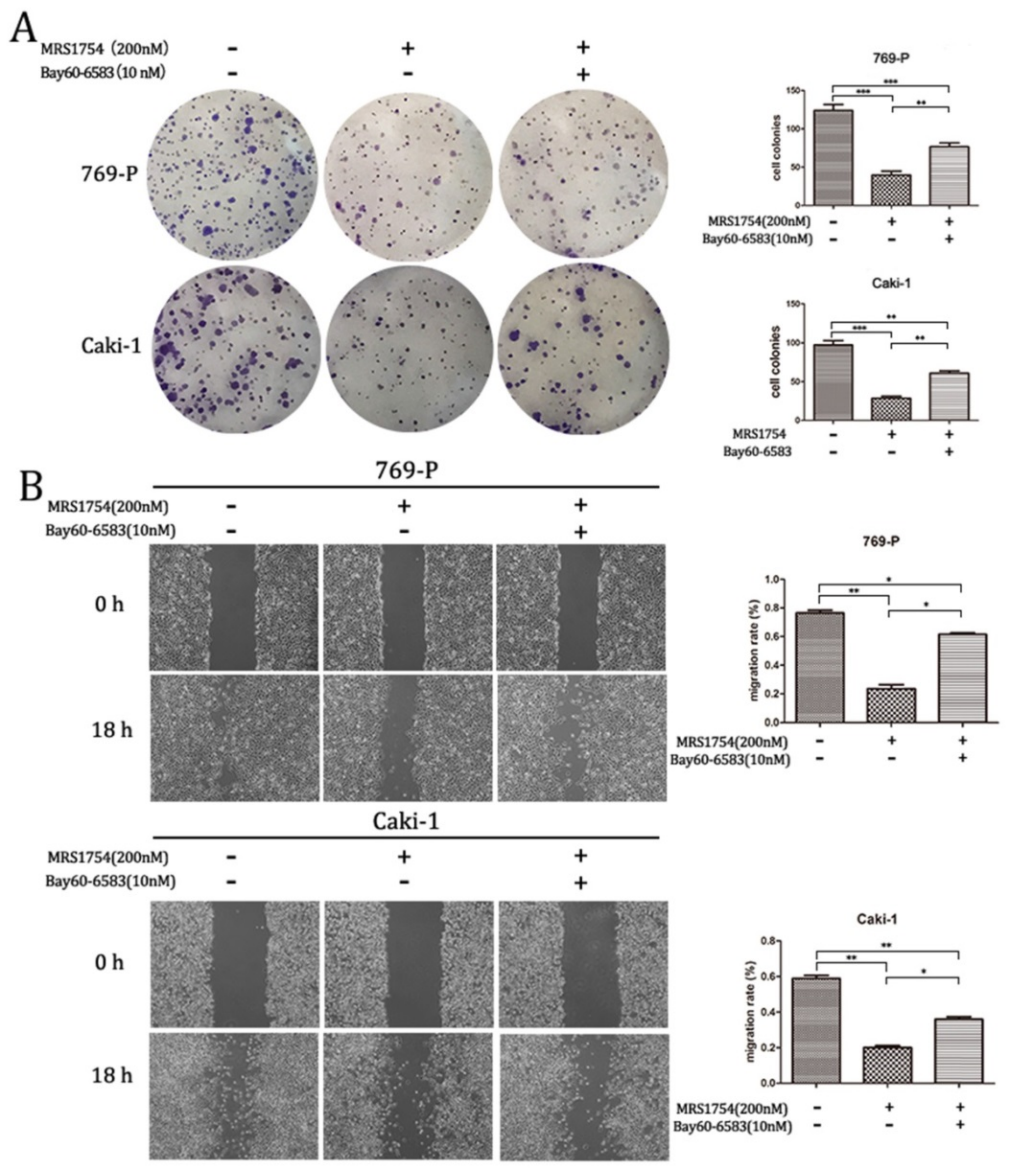
Bay60-6583 rescued the inhibitory effects of MRS1754 on cell growth and migration. (A)Colony formation assay showed Bay60-6583 rescued the MRS1754-induced reduction in cell growth. (B)Wound healing assay showed Bay60-6583 (10 nM) reversed the inhibitory effects of MRS1754 on cell migration capability. ***p<0.001, **p<0.01, *p<0.05.

To further explore the role of A2bR blockade on RCC cells growth and migration, Bay60-6583 (10 nM), a selective A2bR agonist, was used in colony formation assay and wound healing assay. Bay60-6583, the selective A2bR agonist, did not significantly affect cell growth and migration capacity at the concentration of 10 nM (The results aren't shown). However, as shown in Fig. 4, treatment of Bay60-6583 (10 nM) could reverse the inhibitory effect of MRS1754 (200 nM) on RCC cells growth and migration capacity significantly. Together, A2bR played an important role in malignancy and A2bR blockade may be a promising therapy.

### A2bR knockdown inhibits tumor growth in nude mice

**Figure 5:**
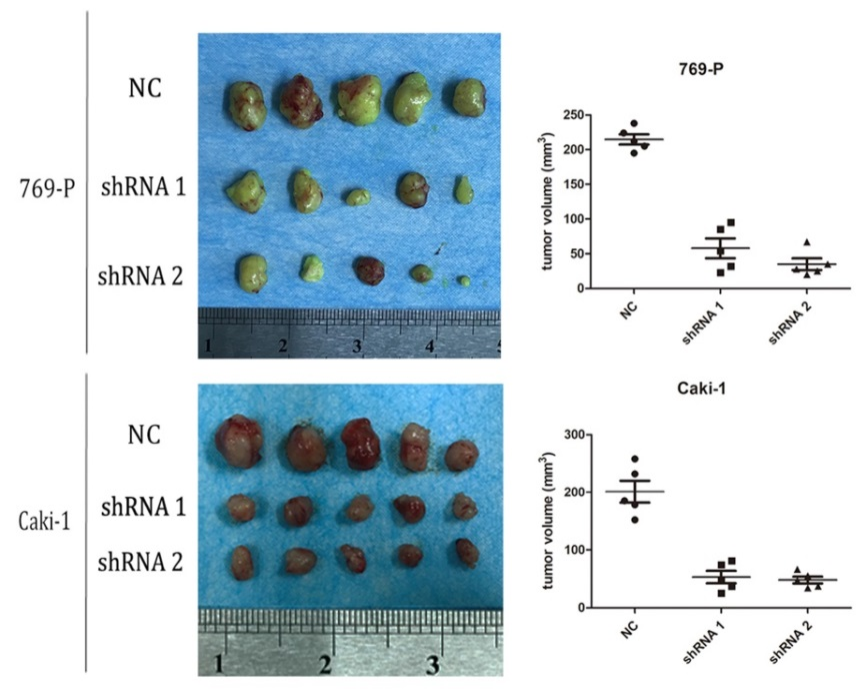
A2bR blockade inhibited cell growth of 769-P and Caki-1 cell lines* in vivo*. Nude mice were sacrificed at the 40th days after subcutaneous injection (n=5 for each group) and tumors were removed. The volume of tumors were measured as width2×length/2.

As mentioned above, we found that blockade of adenosine A2b receptor inhibited cell proliferation of RCC cells *in vitro*. We supposed it would further inhibit tumor growth *in vivo*. To confirm it, 769-P and Caki-1 cells transfected with shRNA-A2bR were injected subcutaneously into BABL/c mice to establish syngeneic tumors. At the 40th day after subcutaneous injection, the mice were sacrificed and tumors were completely removed. We found the average volumes of tumors in shRNA-A2bR group were dramatically decreased, compared with NC group (Fig. 5; *p*<0.05). These data further confirmed that A2bR knockdown could restrain the ability of cell growth in RCC cells.

### A2bR blockade inhibits malignancy of RCC cells via MAPK/JNK signal pathway

**Figure 6:**
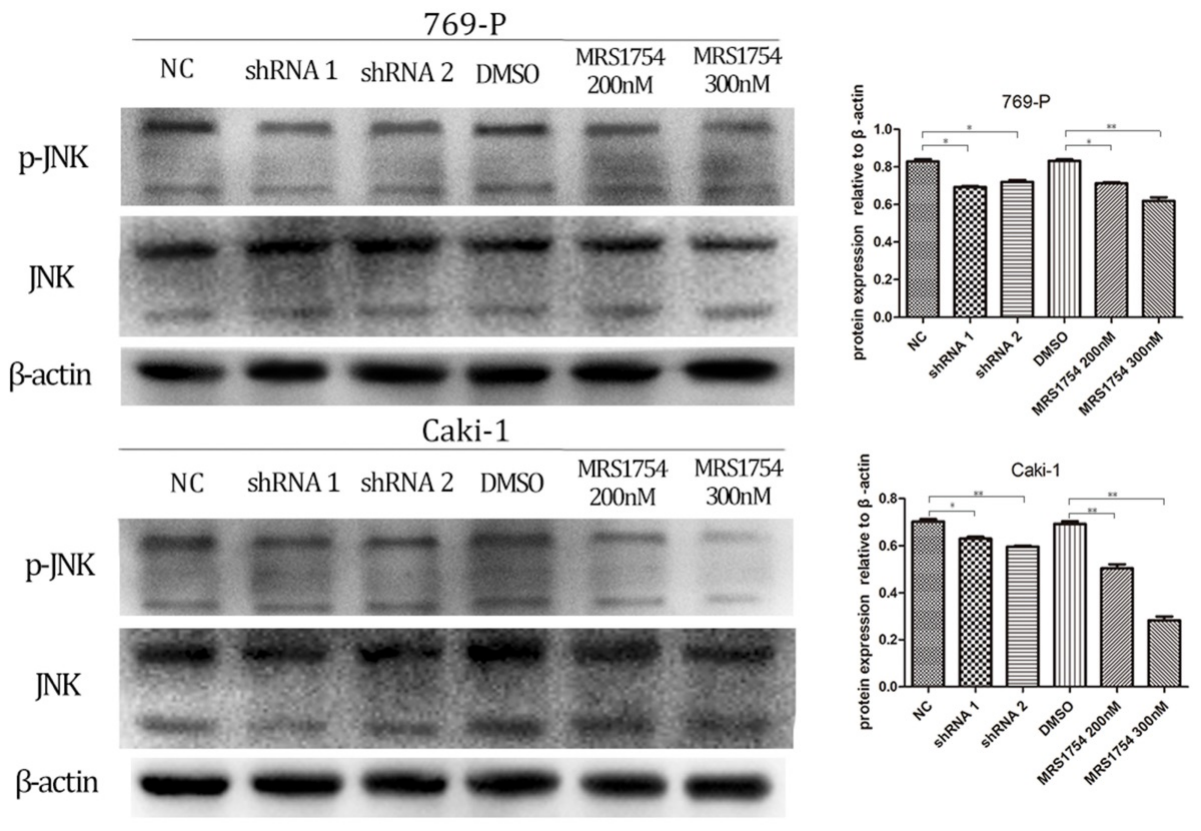
A2bR blockade decreased expression of MAPK/JNK pathway proteins in 769-P and Caki-1 cell lines. Down-regulation of adenosine A2b receptor inhibited the phosphorylation level of MAPK/JNK protein. While MRS1754 inhibited the phosphorylation level of MAPK/JNK protein in a dose-dependent manner.

We next determined the potential mechanism by which A2bR blockade inhibited tumor growth, migration and invasion in 769-P and Caki-1 cells. Previous studied have shown that A2bR activated JNK pathway and exerted its anti-immune function 19. Therefore, we determined whether JNK pathway participated in anti-tumor effects mediated by A2bR blockade in RCC cell lines 769-P and Caki-1. Western blotting showed that MRS1754 significantly decreased the expression of p-JNK in concentration-dependent manners after 2 days of incubation of MRS1754 (Fig. 6;* p*<0.05). Moreover, down-regulation of A2bR also markedly suppressed phosphorylation level of JNK protein (Fig. 6; *p*<0.05).

**Figure 7:**
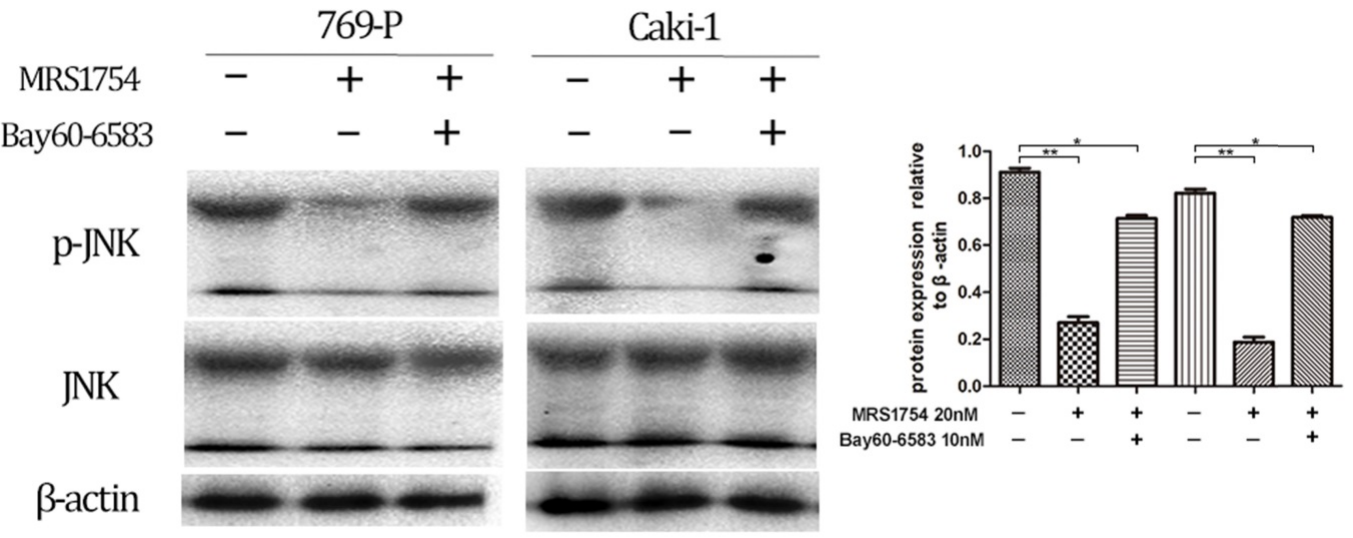
Bay60-6583 could rescue the decreased expression of MAPK/JNK pathway protein in 769-P and Caki-1 cell lines. MRS1754 inhibited the phosphorylation level of MAPK/JNK protein, which could be rescued by Bay60-6583(10nM), an A2bR selective agonist.

In addition, the cells were cultured with MRS1754 (200 nM) with/without treatment of Bay60-6053 (10 nM), which at the concentration of 10nM didn't affect phosphorylation level of JNK protein (The results aren't shown). After 2 days of treatment of both MRS1754 and Bay60-6053, the results showed that Bay60-6053 could rescue the expression of p-JNK protein which was suppressed by MRS1754 (Fig. 7;* p*<0.05). Together, A2bR blockade may mediate the anti-tumor function in part via MAPK/JNK signal pathway in 769-P and Caki-1 cell lines.

## Discussion

The evidence is growing that adenosine acts as a crucial regulatory autocrine and paracrine factor accumulating in the cellular microenvironment 5. The concentration of adenosine, which is at low levels under physiological condition, can rapidly increase in response to certain conditions, such as inflammation, hypoxia, ischemia or trauma 22. Indeed, the rapid accumulations of extracellular adenosine have a protective effect, which prevents cells from excessive inflammatory response, to help tissues restore homeostasis 10, 23. However, in tumor environment, the chronic accumulation of adenosine may be associated with immunosuppression that favors the onset of neoplasia 24. Adenosine, acting as a signaling molecule, binds to adenosine receptors 25. Compared with other adenosine receptors, adenosine A2b receptor (A2bR) shows a lower affinity for adenosine binding. Therefore, A2bR is only stimulated in conditions associated with massive adenosine release, such as tumor hypoxic environment caused by inadequate perfusion of oxygen 11, 26. Studies have described expression of A2b receptor in various tumor cell lines. In colon, prostate, ovarian carcinoma, adenosine A2b receptor is expressed predominantly with respect to other adenosine receptors, which suggests that it may play a role in the pathogenicity and tumor progression. 16, 17, 25, 27. It is well known that most solid tumors do not receive sufficient oxygen and hypoxia is commonly observed in their tumor microenvironment 16. Renal cell carcinoma, a typical solid tumor, is common in urologic tumor. We hypothesized that adenosine A2b receptor may be linked to renal cell carcinoma progression. However, the expression and function of A2bR in renal cell carcinoma cells remains unclear. To our knowledge, the current study demonstrates for the first time that selective A2bR blockade can reduce growth, migration and invasion capacity of renal cell carcinoma 769-P and Caki-1 cell lines. Multiple mechanisms are involved in this progression, such as regulation of immune system, angiogenesis and direct effects on cancer cells 23, 28. This study focused on the direct effects of adenosine on cancer cell progression.

In this study, we firstly detected the effects of MRS1754, a selective A2bR antagonist, on proliferation pattern of cells. We observed that the cell growth of 769-P and Caki-1 was significantly decreased in dose-dependent manners with the treatment of MRS1754. Furthermore, we tested the effects of inhibition by MRS1754 on migration and invasion capacity of cancer cells. Our data showed that the migration and invasion pattern of renal cell carcinoma cells were also restrained with the treatment of MRS1754. Moreover, we assessed the MAPK/JNK pathways, which were up-regulated frequently in various cancers. And our results showed that MRS1754 significantly decreased phosphorylation levels of JNK protein with treatment of MRS1754. It had been established that MRS1754 could inhibit tumor patterns of 769-P and Caki-1 cell lines; however, it was not clear whether MRS1754 affected the expression of A2bR. Typically, pharmacologic inhibit the active form of the receptor, but not change the receptor expression. To clarify it, we used different concentrations of MRS1754 to treat cells for 5 days. The, the mRNA and protein expression results showed MRS1754 had no significant effects on adenosine A2b receptor expression, which conforms to the previous theory. In addition, we transfected 769-P and Caki-1 cell lines with shRNA-A2bR to knock down the expression of adenosine A2b receptor. As expected, the biological effects of tumor cells were significantly restrained compared with NC groups. Together, the results showed that A2bR blockade inhibited growth, migration and invasion patterns of renal cell carcinoma cell lines in part by suppressed MAPK/JNK pathway.

The ability to proliferate without limit is a major characteristic of tumors. Adenosine A2b receptor has been reported to play important roles in cell proliferation. A2bR-positive oral squamous cell carcinoma-derived cells were correlated closely with tumoral size and suppression of A2bR expression with shRNA significantly inhibited cellular proliferation compared with the control groups 29. Furthermore, adenosine A2b receptor blockade could decrease VEGF secretion in melanoma cells to promote chemotherapy-induced apoptosis by impairing IL-8 production 30. In colonic carcinoma cells, the neoplastic growth was delayed by inhibition of A2bR using MRS1754 16. Studies above suggested that adenosine A2b receptor may have cancer-promoting properties. In addition, recent studies showed that adenosine A2b receptor is coupled to Gs protein, which induces an increase in cAMP levels, followed CREB activation by PKA, thereby increasing cell proliferation 31. Based on these findings, we observed that adenosine A2b receptor was highly expressed in RCC 769-P and Caki-1 cell lines. As expected, pharmacological and genetic blockade of A2bR was found to inhibit cellular proliferation *in vitro* and the growth of tumor *in vivo*. Taken together, our results suggest that adenosine A2b receptor blockade may inhibit renal cell carcinoma growth.

Migration and invasion are the most vital properties of malignant tumors and also are the ultimate causes of death in cancer patients 32. Accumulating evidence highlights a crucial involvement of CD73 in the modulation of metastatic processes 33. The contribution of CD73 to metastasis has focused on cancer cells' adhesion to the extracellular matrix and invasiveness 34, 35. Meanwhile, increasing evidence suggests CD73-derived adenosine also enhances tumor cell migration via A2bR 36. The expression of A2bR in metastatic colorectal cancer cell line (SW620) is significantly higher in non-metastatic model (SW480) 37. Both RNAi silencing and pharmacologic blockade of A2bR inhibited invasive activity of breast cancer cells and correspondingly reduced tumor outgrowth in the lungs 38. Moreover, in mouse models of melanoma and triple-negative breast cancer metastasis, adenosine A2b receptor inhibitor significantly decreased metastasis *in vivo* and *in vitro* 39. Ntantie et al 40 have provided a detailed mechanism that A2bR suppressed the prenylation of small GTPase RAS-related protein1, which contributed to formation and maintenance of cell adherent junction, thereby leading to an increase of cell scattering. Consistent with these studies, our results also showed that A2bR blockade was capable of inhibiting the migration and invasion in 769-P and Caki-1 cells.

Furthermore, it has been reported that transcription factor Fra-1 was linked to tumor cells metastatic ability, which is related to epithelial phenotype to mesenchymal phenotype transition 41, 42. There are no drugs that regulate Fra-1 activity, but the gene that regulates Fra-1 is the adenosine A2b receptor 19, 41. Interestingly, MAPK/Fra-1 pathway has been reported to modulate tumor formation 43. Therefore, we further determined whether adenosine A2b receptor modulated biological effects of RCC cell lines 769-P and Caki-1 via MAPK pathways. Consistent with previous studies, the results of western blotting showed that adenosine A2b receptor blockade significantly reduced the phosphorylation level of JNK signaling pathway. Taken together, our study demonstrated that A2bR blockade inhibit the cell proliferation, migration and invasion ability in part via modulating the MAPK/JNK pathway.

In summary, we have demonstrated the expression of A2bR in RCC cell lines 769-P and Caki-1 and its biological effects on proliferation, migration and invasion of renal cell carcinoma, suggesting that development of effective adenosine A2b receptor blockade would be a practical approach for renal cell carcinoma.

## Figures and Tables

**Table 1:** List of primers used in RT-PCR.

Target	Primer sequence
A2bR	F:5'- ATCCCATTGTCTATGCTTACCG -3'
	R:5'- CATTCCCACTCTTGACATCTGC -3'
β-actin	F:5'- TGACGTGGACATCCGCAAAG -3'
	R:5'- CTGGAAGGTGGACAGCGAGG -3'
